# Clean Intermittent Catheterization in Children under 12 Years Does Not Have a Negative Impact on Long-Term Graft Survival following Pediatric Kidney Transplantation

**DOI:** 10.3390/jcm13010033

**Published:** 2023-12-20

**Authors:** Marios Marcou, Matthias Galiano, Anja Tzschoppe, Katja Sauerstein, Sven Wach, Helge Taubert, Bernd Wullich, Karin Hirsch-Koch, Hendrik Apel

**Affiliations:** 1 Clinic of Urology and Pediatric Urology, University Hospital Erlangen, Friedrich-Alexander-Universität Erlangen-Nürnberg, 91054 Erlangen, Germany; sven.wach@uk-erlangen.de (S.W.); helge.taubert@uk-erlangen.de (H.T.); bernd.wullich@uk-erlangen.de (B.W.); karin.hirsch@uk-erlangen.de (K.H.-K.); hendrik.apel@uk-erlangen.de (H.A.); 2 Clinic of Pediatrics and Adolescent Medicine, University Hospital Erlangen, Friedrich-Alexander-Universität Erlangen-Nürnberg, 91054 Erlangen, Germany; matthias.galiano@uk-erlangen.de (M.G.); anja.tzschoppe@uk-erlangen.de (A.T.); katja.sauerstein@uk-erlangen.de (K.S.); 3 Transplantation Center Erlangen-Nürnberg, University Hospital Erlangen, Friedrich-Alexander-Universität Erlangen-Nürnberg, 91054 Erlangen, Germany

**Keywords:** child, kidney transplantation, catheterization, CAKUT, LUTD

## Abstract

Background: Congenital anomalies of the kidneys and urinary tract (CAKUTs) are one of the most prevalent primary causes of end-stage renal disease (ESRD) in young children, and approximately one-third of these children present with lower urinary tract dysfunction (LUTD). Many children with LUTD require therapy with clean intermittent catheterization (CIC). CIC commonly leads to bacteriuria, and considerations have arisen regarding whether CIC in immunosuppressed children is safe or whether repeated febrile urinary tract infections (UTIs) may lead to the deterioration of kidney graft function. Material and Methods: We retrospectively reviewed all cases of primary kidney transplantation performed in our center between 2001 and 2020 in recipients aged less than twelve years. The number of episodes of febrile UTIs as well as the long-term kidney graft survival of children undergoing CIC were compared to those of children with urological causes of ESRD not undergoing CIC, as well as to those of children with nonurological causes of ESRD. Results: Following successful kidney transplantation in 41 children, CIC was needed in 8 of these patients. These 8 children undergoing CIC had significantly more episodes of febrile UTIs than did the 18 children with a nonurological cause of ESRD (*p* = 0.04) but not the 15 children with a urological cause of ESRD who did not need to undergo CIC (*p* = 0.19). Despite being associated with a higher rate of febrile UTIs, CIC was not identified as a risk factor for long-term kidney graft survival, and long-term graft survival did not significantly differ between the three groups at a median follow-up of 124 months. Conclusions: Our study demonstrates that, under regular medical care, CIC following pediatric transplantation is safe and is not associated with a higher rate of long-term graft loss.

## 1. Introduction

Congenital anomalies of the kidneys and urinary tract (CAKUTs) are one of the most prevalent primary causes of end-stage renal disease (ESRD) in young children [1], and approximately one-third of these children present with lower urinary tract dysfunction (LUTD) [2,3,4]. Patients with LUTD show an increased incidence of urinary tract infections (UTIs) and symptomatic high-pressure vesicoureteral reflux (VUR) [5]. Left untreated, following renal transplantation, LUTD will lead to a deterioration of kidney graft function, and the kidney graft will suffer the same terminal fate as the native kidneys of these patient [6], making repeated evaluations of these patients prior to and after kidney transplantation imperative [7]. Depending on the severity of bladder dysfunction, the management strategies for such cases range from simple medication therapy to surgical therapy up to a lifelong recommendation for clean intermittent catheterization (CIC) [8,9].

CIC, the repeated insertion of a catheter to drain urine from the bladder, has shown significant advantages in comparison to indwelling catheters, and guidelines recommend CIC over continuous catheterization [10]. Besides an improved quality of life and improved sexuality in adult life, CIC is thought to cause fewer UTIs, fewer urethral complications, less stone disease and less upper urinary tract damage, and regular health care visits for catheter changes are not required, compared to when applying indwelling catheters [11].

CIC commonly leads to bacteriuria [12]. Bacteriuria combined with immunosuppression following pediatric kidney transplantation is assumed to be a risk factor for the development of febrile UTIs or could even lead to sepsis, which could affect long-term graft survival or even patient survival [13,14,15]. In our study, we retrospectively reassessed all cases of children under twelve years of age undergoing CIC following pediatric kidney transplantation in our center and asked, from a retrospective point of view, whether CIC had a possible impact on the long-term graft survival or the overall patient survival of young child recipients.

## 2. Materials and Methods

We retrospectively reviewed all cases of primary kidney transplant in recipients aged less than twelve years, performed in our center between 2001 and 2020. Cases of retransplantation following graft failure after pediatric kidney transplantation in the past were excluded from the study. Using our hospital database, the electronic medical files of all primary cases of pediatric kidney transplantation were thoroughly investigated, and children under twelve years of age undergoing CIC posttransplantation were identified. These children under 12 years of age are part of a previously described pediatric kidney transplantation cohort [16]. The clinical characteristics of this cohort are described in Table 1 and Table 2. The primary outcome of this study was graft survival, determined as the need to restart dialysis, transplantectomy or retransplantation. The secondary outcome of the study was febrile UTIs, defined as a positive urine culture accompanied by fever, where all other causes of febrile infection had been ruled out. The characteristics were compared by the two-sample t test and Mann—Whitney U test, and graft survival estimates were analyzed using the Kaplan—Meier method. Statistical analysis was performed using the R statistical computing program (version 4.2.1), with statistical significance set at *p* < 0.05 [17].

## 3. Results

Between 2001 and 2020, a total of 44 primary kidney transplantations in children under the age of twelve years were undertaken in our center. In this designated time period, no significant changes in the management of pediatric kidney transplant recipients were undertaken in our center, and no significant variations in data distribution between the first and the second decade of the study were observed. The median age of the children was six years (IQR 5–8 years). The main causes of ESRD in the children in our study were posterior urethral valves (PUVs) (29.5%), followed by renal dysplasia (15.9%) and reflux nephropathy (13.6%), as shown in Table 1. Forty-one of the forty-four grafts (93%) originated from a deceased donor (DD), and only three (7%) originated from a living donor (LD). Following transplantation, early graft failure was observed in 3 of the 44 cases (7%), 1 of which was an LD transplantation.

In 23 (19 boys and 4 girls) of the remaining 41 children with a functioning graft, a urological LUTD background for ESRD was observed (posterior urethral valves in 13 boys, reflux nephropathy, VACTERL association, high-confluence urogenital sinus, etc.). In 8 of the 23 cases (35%) with LUTD, including seven boys and one girl, CIC was needed and consequently undertaken, initially through the caregivers of the affected children and eventually through the affected children themselves. The baseline characteristics of the children in every group are listed in detail in Table 2.

All children with symptomatic vesicoureteral reflux (VUR) in the native kidneys were treated prior to kidney transplantation. Children with persistent high-grade reflux in the native kidneys were treated with nephrectomy prior, during or shortly after kidney transplantation, and in some cases, a megaureter was used for augmenting the bladder. Bladder augmentation because of a small, low-compliance bladder was necessary in 6 of the 23 patients with LUTD. In five of these six patients, ureterocystoplasty with the use of a native megaureter following nephrectomy of one of the native kidneys was performed, while the other remaining patient underwent enterocystoplasty. Following cystoplasty, CIC was needed in all six cases. In only one of the six cases that underwent cystoplasty, catheterization continued to be performed transurethrally, while in the other five cases, a continent vesicostomy was created. In three of the five cases, a vesicostomy was performed with the use of a megaureter stump following nephrectomy of one of the native kidneys, and in the other two cases, an appendicovesicostomy (Mitrofanoff) was performed. An isolated continent vesicostomy, without the necessity of augmenting the bladder, was needed in two cases: one was performed with the use of a megaureter following nephrectomy of a native kidney and one with the use of the appendix (Mitrofanoff). Two of the abovementioned procedures (a ureterocystoplasty with the creation of a Mitrofanoff stoma and the formation of an isolated continent vesicostomy with the use of a megaureter) were performed during renal transplantation. The remaining procedures were performed in the weeks and months following renal transplantation.

The median follow-up of the eight children requiring CIC was 135 months, while it was 114 months for children without LUTD and 126 months for children with LUTD who did not need to undergo CIC. During follow-up, a median of 2.5 febrile UTIs were documented for every patient in the CIC group compared to a median of only one febrile UTI in the group with no LUTD and of two febrile UTIs in the group of children with LUTD who did not need to undergo CIC (*p* = 0.045), as reported in Table 2. Following the first episode of a febrile UTI, a diagnostic reevaluation of the patient, including a voiding cystourethrography, was always performed, and a VUR in the transplanted kidney was revealed in a total of six patients (four children without LUTD, one child with LUTD who did not need to perform CIC and one child undergoing CIC). VUR in the transplanted kidney was treated successfully endoscopically in four of the six cases, while an open anti-reflux procedure was required in the remaining two cases (in the case of the child with LUTD undergoing CIC and in the case of a child without LUTD). A statistical analysis did not reveal a significant association of VUR in the transplanted kidney with a poorer long-term kidney graft survival (*p* = 0.112). Antibiotic prophylaxis was only administrated following an episode of febrile UTI and was usually discontinued in the months thereafter, following a diagnostic reevaluation of the patient and after necessary adaptations in therapy had taken place or following the treatment of a VUR in the transplanted kidney.

**Figure 1:**
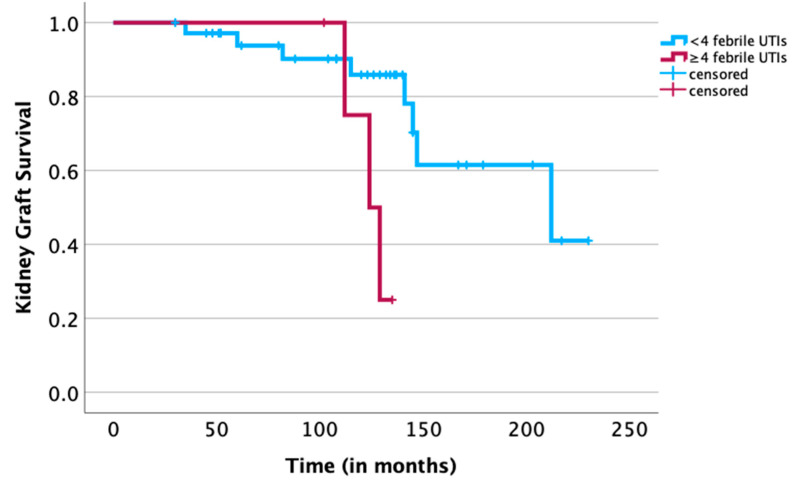
Survival analysis using the Kaplan—Meier method for recipients following pediatric kidney transplantation with at least four febrile UTIs (≥4 febrile UTIs, *n* = 5) in a total of 41 recipients with a functioning graft. A univariate log-rank analysis revealed a significant difference in long-term kidney graft survival between the two groups (*p* = 0.026) (censored for end of follow-up).

A univariate analysis of the baseline characteristics of all recipients (Table 2) identified the age of the organ donor to be a negative predictive factor for organ failure (HR = 1.063 per year of donor age, *p* = 0.018), as shown in Table 3. Furthermore, in univariate analyses, the number of febrile UTIs during follow-up was confirmed to be a negative predictor of graft survival (HR = 1.220, *p* = 0.026), as shown in Table 3. Stratifying febrile UTIs into absolute occurrences of a febrile UTI revealed a cutoff of at least four febrile UTIs (≥4) as statistically associated with graft survival (*p* = 0.026, Figure 1). In a multivariate analysis, corrected for the two significant predictors of graft failure, age of the donor and number of febrile UTIs, the grouping exhibited no predictive properties (Table 4).

**Figure 2:**
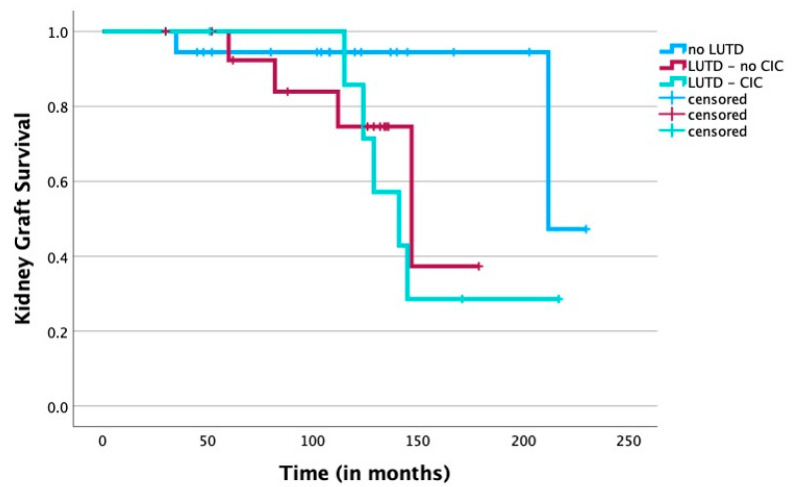
Survival analysis for kidney grafts using the Kaplan—Meier method for recipients undergoing CIC compared to children in the no-LUTD group as well as to children with LUTD that did not require CIC. A univariate log-rank analysis did not show any statistically significant difference between the three groups (*p* = 0.08) (censored for end of follow-up).

The kidney graft survival rates in the CIC group at 5 and 10 years were 100% and 85.7%, respectively, compared to 92.3% and 74.6% for children with LUTD that did not require CIC and 94.4% after 5 as well as after 10 years for the no-LUTD group. A log-rank survival analysis using the Kaplan—Meier method revealed no significant difference in long-term graft survival among the three groups (Figure 2). No death of the recipients was documented during follow-up.

## 4. Discussion

Since its introduction in 1972 [18], CIC has become the standard therapy for a number of bladder function disorders (LUTDs). These include incomplete bladder voiding because of an underactive or atonic bladder, such as in the case of myogenic failure in patients with congenital PUV, detrusor–sphincter dyssynergia or a low-compliance bladder causing high intravesical pressure, where medication therapy or sometimes augmentation of the bladder is needed [19]. Left untreated, all the above-mentioned conditions can lead to hydroureteronephrosis and UTIs and are known to greatly contribute to renal function deterioration, being very often the underlying cause of ESRD in children [20,21]. CIC has been shown to stop renal deterioration in patients with LUTD and is in some cases necessary following pediatric kidney transplantation. Without CIC, the transplanted kidney will suffer the same consequences as the native kidneys [6]. CIC has proven to be a safe and easy-to-perform procedure. The caregivers of the affected children as well as the children themselves have proven, in their majority, to be fast and excellent learners of CIC [22]. CIC offers unique advantages when compared to incontinent urinary diversions or indwelling catheters, with such advantages being a higher quality of life, better social adaptation, a higher self-esteem of the children and improved sexuality in adulthood [23].

A number of epidemiological studies have shown that congenital anomalies of the kidneys and the urinary tract (CAKUT) are among the most common causes of ESRD in children [1]. This was also confirmed in our study, and the identification of LUTD prior to kidney transplantation is of the utmost importance. Meticulous repeated ultrasound studies of the kidneys and urinary tract as well as repeated video urodynamic studies of LUTD patients are essential in planning and continuously adapting the therapy of these patients before and after kidney transplantation [7,24].

Interestingly, in our group of kidney graft recipients, LUTD was present in 19 males and only 4 females. Posterior urethral valves (PUVs), a major cause of LUTD and ESRD in children, provoke a disease that affects only males [25]. Further analysis of the baseline characteristics of all the children in our study once again confirmed a significant association between donor age and long-term graft survival. This was demonstrated in a series of studies [26,27,28], and our study group previously reported on these results [16]. Although some of the remaining baseline characteristics, such as human leucocyte antigen (HLA) mismatches and kidney graft cold ischemia time, were, in other studies, repeatedly demonstrated to play a significant role in long-term graft survival [29,30,31,32,33,34], our study did not reveal any significant association between any of these factors and long-term kidney graft survival. The number of HLA mismatches exceeded four in only two of the 41 children in our study, and the cold ischemia of the kidney graft exceeded twelve hours in only five cases, showing that in our center, an extremely careful selection of deceased donor organs took place in the decision-making process during organ allocation.

In our study, all augmentation procedures or procedures for the creation of a continent vesicostomy were performed following successful transplantation or, in two cases, during kidney transplantation. Although several studies reported equally good results with augmentation of the bladder prior to transplantation [35], the overwhelming percentage of DD-kidney transplantations in our center did not allow us to safely plan operations prior to transplantation. In addition, concern regarding an increasing wait list time and fear of possible complications because of anuria led us to postpone surgical interventions for the treatment of LUTD until after successful DD kidney transplantation had taken place. Of the eight patients in need of CIC, six underwent augmentation of the bladder (ureterocystoplasty in five cases and enterocystoplasty in one case), and in seven patients, a continent vesicostomy was created (with the use of a ureteral stump in five cases and of a Mitrofanoff stoma in two cases). Augmentation of the bladder was significantly associated with a higher rate of febrile UTIs (*p* = 0.008) but not with a poorer long-term kidney graft survival (*p* = 0.2). Although the indication for augmentation of the bladder was always based on strict objective criteria, such as high detrusor pressures at reduced bladder capacity and incontinence resulting from idiopathic detrusor instability [36], and the procedure was performed only after exhausting all other therapeutic possibilities, the indication for the creation of a continent vesicostomy was sometimes based on subjective criteria provided by the child or the caregivers of the child, and a vesicostomy was usually created to ease catheterization. In our study, the creation of a vesicostomy was statistically associated neither with a higher rate of febrile UTIs (*p* = 0.088) nor with a poorer long-term kidney graft survival rate (*p* = 0.3). There is, however, no evidence that catheterization via a stoma can reduce UTI episodes in patients undergoing CIC [37].

Although CIC is associated with significantly fewer complications than indwelling catheters [38], asymptomatic bacteriuria in patients undergoing CIC is common [12], and considerations regarding the risk and impact of repeated UTIs, especially in immunosuppressed children, have emerged repeatedly over time [3,39]. In accordance with previous publications, the number of febrile UTIs in our study was associated with poorer long-term kidney graft survival (Table 3). Other studies also demonstrated that recurrent febrile UTIs in pediatric kidney recipients may lead to faster graft deterioration [40], and in our study, a cutoff of at least four febrile UTIs was significantly associated with worse kidney graft survival (*p* = 0.026, Figure 1). However, these cases were not limited to the group of children undergoing CIC, and CIC was not identified as an independent risk factor for long-term kidney graft survival. We suggest that through the regular medical reevaluations of children undergoing CIC, as well as through continuous education and support of these children and their caregivers provided by a specialized team in our center, it became possible to eventually limit but, sadly, not completely eliminate complications such as febrile UTIs. Although the number of febrile UTIs among children undergoing CIC was significantly higher than in other children and the number of UTIs was associated with a worse graft survival, CIC was not found to be an independent predictor of renal graft failure. Our findings are also supported by other reports that indicated that, despite acute graft dysfunction during episodes of a febrile UTI, the long-term renal function ultimately did not differ between patients with and without infection [3,41].

Despite the fact that previous studies postulated that transplantation into the dysfunctional lower urinary tract is associated with high complication rates and inferior graft survival [42,43], more contemporary studies demonstrated comparable results between children with primary urological abnormalities and those with nonurological abnormalities [15,21,24,44,45]. However, considerations, also in contemporary studies, regarding whether a dysfunctional bladder may contribute to graft deterioration in the long term has arisen [46]. Our study demonstrates that transplantation in children with LUTD is safe and that CIC in particular does not have a negative impact on long-term kidney graft survival. To our knowledge, our study is the first to investigate the implications of CIC in small children under twelve years of age, with a long-term follow-up of the patients.

The main limitations of our study are its small size, which is reflective of the low incidence of kidney transplantation in children under twelve years and the even lower incidence of CIC necessity in these children, as well as its retrospective design. The small number of subjects in some subgroups, such as that of patients undergoing transurethral catheterization versus that of patients subjected to catheterization via stoma or that of pediatric kidney recipients after ureterocystoplasty versus that of recipients after enterocystoplasty, prohibited a comprehensive investigation of these subgroups. Furthermore, our study did not take into consideration events following transplantation that could very well influence long-term kidney graft survival, such as the immunotherapy schemes used or future infections, as well as the ultimate reason for graft failure. It is our opinion that large multicenter prospective studies are needed to finally cast away any shadow of possible risks attached to CIC, and we hope that our data will help to raise the acceptance of CIC following pediatric kidney transplantation.

## 5. Conclusions

Following successful transplantation in forty-one children under the age of twelve, CIC was necessary in eight children. Although these children undergoing CIC had significantly more episodes of febrile UTIs compared to the eighteen children with a nonurological cause of ESRD, the long-term graft survival did not significantly differ between the two groups at a median follow-up of 124 months. Despite its small size, our study demonstrated that, under regular medical care, transplantation in young children with severe LUTD requiring CIC is equally safe and effective as kidney transplantation in children with a normal lower urinary tract.

## Figures and Tables

**Table 1:** Primary causes of end-stage renal disease (ESRD) in children under twelve years having undergone primary kidney transplantation in the last twenty years in our center.

Causes of ESRD in Children <12 Years Old in Our Center	*n* = 44
Posterior urethral valves (PUVs)	13 (29.5%)
Renal aplasia/hypoplasia/dysplasia	7 (15.9%)
Reflux nephropathy	6 (13.6%)
VACTERL association	4 (9.0%)
Focal segmental glomerulosclerosis (FSGS)	2 (4.5%)
Congenital nephrotic syndrome	2 (4.5%)
Hemolytic uremic syndrome	2 (4.5%)
Polycystic kidney disease	2 (4.5%)
Other (cystinosis, bilateral nephrectomy, etc.)	6

**Table 2:** Baseline characteristics of children under the age of 12 who underwent primary pediatric transplantation in our center between 2001 and 2020. In the table, it is made obvious that more males than females had lower urinary tract dysfunction (LUTD). Posterior urethral valve (PUV) disorder, a major cause of LUTD and ESRD in children, is a disease that affects only males. Of the 44 children under the age of 12 that were transplanted in our center between 2001 and 2020, 3 with early graft failure were excluded from the study (significant values are marked in boldface).

Parameter	Control Group (No LUTD) *n* = 18	LUTD CIC Group *n* = 8	LUTD No CIC *n* = 15	*p*
Organ donation				**0.013**
Living donation	0	2	0	
Postmortem donation	18	6	15	
Recipient sex				**0.036**
Male	8	7	12	
Female	10	1	3	
Age of the recipient (years; median (IQR))	7 (5–8)	7 (4–10.5)	6 (5–7)	0.527
Age of the donor (years; median (IQR))	32 (16–51)	43 (35–53)	34 (20–46)	0.475
BSA of the recipients at transplant (m^2^; median (IQR))	0.84 (0.72–0.93)	0.89 (0.71–1.14)	0.80 (0.70–0.83)	0.492
Pretransplant time of the recipient on dialysis (months; median (IQR))	24 (15–33)	21 (11–34)	41 (21–53)	0.105
HLA mismatches (*n*; median (IQR))	3 (2–3)	3 (2–4)	2 (2–3)	0.652
Cold ischemia time of the graft (h; median (IQR))	12 (10–16)	14 (8–17)	12 (11–15)	0.930
Urinary tract infections (*n*; median (IQR))	1 (0–2)	2.5 (2–4)	2 (0.5–3)	**0.045**
Follow-up of kidney grafts (months; median (IQR))	114 (86–144)	135 (122–152)	126 (72–135)	0.420

Abbreviations: BSA, body surface area; CIC: clean intermittent catheterization; LUTD, lower urinary tract dysfunction.

**Table 3:** Univariate Cox regression survival analysis of risk factors for long-term kidney graft survival following pediatric kidney transplantation. The age of the organ donor and the number of febrile UTIs during follow-up were identified to be negative predictors of graft survival (significant values are marked in boldface).

Parameter	HR (95% CI)	*p*
Organ donation		
Living donation	1 (Reference)	
Postmortem donation	1.103 (0.132–9.235)	0.928
Recipient sex		
Female	1 (Reference)	
Male	2.587 (0.551–12.160)	0.229
Age of the recipient (years; median (IQR))	0.928 (0.738–1.167)	0.524
Age of the donor (years; median (IQR))	1.063 (1.011–1.119)	**0.018**
BSA of the recipients at transplant (m^2^; median (IQR))	0.775 (0.021–28.18)	0.889
Pretransplant time of the recipient on dialysis (months; median (IQR))	1.011 (0.989–1.042)	0.489
HLA mismatches (*n*; median (IQR))	1.004 (0.503–1.901)	0.990
Cold ischemia time of the graft (h; median (IQR))	1.013 (0.909–1.130)	0.813
Urinary tract infections (*n*; median (IQR))	1.220 (1.024–1.454)	**0.026**
Grouping		
Control (no LUTD)	1 (Reference)	
LUTD CIC group	4.091 (0.789–21.200)	0.093
LUTD no CIC	3.197 (0.558–18.320)	0.192

Abbreviations: BSA, body surface area; CIC, clean intermittent catheterization; LUTD, lower urinary tract dysfunction.

**Table 4:** Multivariate Cox regression survival analysis of risk factors for long-term kidney graft survival following pediatric kidney transplantation. In a multivariate analysis, corrected for the two significant predictors of graft failure—age of the donor and number of febrile UTIs—the grouping exhibited no predictive properties.

Parameter	HR (95% CI)	*p*
Age of the donor (years; median (IQR))	1.066 (0.999–1.136)	0.051
Urinary tract infections (*n*; median (IQR))	1.192 (0.978–1.454)	0.083
Grouping		
Control (no LUTD)	1 (Reference)	
LUTD CIC group	0.978 (0.125–7.659)	0.983
LUTD no CIC	2.222 (0.357–13.834)	0.392

## Data Availability

All data are available in the manuscript. Detailed datasets used and analyzed during the present study are available from the corresponding author upon reasonable request.
